# Modeling of Fabry disease nephropathy using patient derived human induced pluripotent stem cells and kidney organoid system

**DOI:** 10.1186/s12967-023-03992-0

**Published:** 2023-02-22

**Authors:** Sheng Cui, Xianying Fang, Hanbi Lee, Yoo Jin Shin, Eun-Sil Koh, Sungjin Chung, Hoon Suk Park, Sun Woo Lim, Kang In Lee, Jae Young Lee, Chul Woo Yang, Byung Ha Chung

**Affiliations:** 1 Transplantation Research Center, Seoul St. Mary’s Hospital, College of Medicine, The Catholic University of Korea, 222 Banpo-daero, Seocho-Gu, Seoul, 06591 South Korea; 2 Division of Nephrology, Department of Internal Medicine, Seoul St. Mary’s Hospital, The College of Medicine, The Catholic University of Korea, 222 Banpo-daero, Seocho-Gu, Seoul, 06591 South Korea; 3 Division of Nephrology, Department of Internal Medicine, Yeouido St. Mary’s Hospital, The College of Medicine, The Catholic University of Korea, Seoul, South Korea; 4 Division of Nephrology, Department of Internal Medicine, Eunpyeong St. Mary’s Hospital, The College of Medicine, The Catholic University of Korea, Seoul, South Korea; 5 ToolGen, Inc, Seoul, Korea

**Keywords:** Fabry disease, hiPSC, Kidney organoids, Disease modeling

## Abstract

**Objectives:**

To explore the possibility of kidney organoids generated using patient derived human induced pluripotent stem cells (hiPSC) for modeling of Fabry disease nephropathy (FDN).

**Methods:**

First, we generated hiPSC line using peripheral blood mononuclear cells (PBMCs) from two male FD-patients with different types of *GLA* mutation: a classic type mutation (CMC-Fb-001) and a non-classic type (CMC-Fb-003) mutation. Second, we generated kidney organoids using wild-type (WT) hiPSC (WTC-11) and mutant hiPSCs (CMC-Fb-001 and CMC-Fb-003). We then compared alpha-galactosidase A (α-GalA) activity, deposition of globotriaosylceremide (Gb-3), and zebra body formation under electromicroscopy (EM).

**Results:**

Both FD patients derived hiPSCs had the same mutations as those detected in PBMCs of patients, showing typical pluripotency markers, normal karyotyping, and successful tri-lineage differentiation. Kidney organoids generated using WT-hiPSC and both FD patients derived hiPSCs expressed typical nephron markers without structural deformity. Activity of α-GalA was decreased and deposition of Gb-3 was increased in FD patients derived hiPSCs and kidney organoids in comparison with WT, with such changes being far more significant in CMC-Fb-001 than in CMC-Fb-003. In EM finding, multi-lammelated inclusion body was detected in both CMC-Fb-001 and CMC-Fb-003 kidney organoids, but not in WT.

**Conclusions:**

Kidney organoids generated using hiPSCs from male FD patients might recapitulate the disease phenotype and represent the severity of FD according to the *GLA* mutation type.

**Supplementary Information:**

The online version contains supplementary material available at 10.1186/s12967-023-03992-0.

## Introduction

Fabry disease (FD) is a glycoshingolipid lysosomal storage disorder resulting from mutational deficiency of α-galactosidase A (α-GalA) enzyme. It is characterized by progressive intracellular accumulation of globotriaosylceramide (Gb-3) in various types of cells including podocytes, renal tubular epithelial cells, and vascular endothelial cells [1]. Progressive accumulation of Gb-3 can result in dysfunction of target organs including kidney. To understand the pathogenesis of Fabry disease nephropathy (FDN) and to develop new drugs, in vivo and also in vitro models of FDN have been established. As an animal model, *GLA* (−/−) mice have provided several important insights into the role of Gb-3 accumulation in the progression of FDN [2–4]. However, animal experiments are not suitable for large-scale screening as they are not genetically identical to humans. Several in vitro models have been introduced through treatment with Gb-3, lyso-Gb-3, or knock-out of *GLA* gene using CRISPR/Cas9 [5–7]. However, these models also have limitation to represent complex cell types comprising the kidney. Recently, zebrafish were found to mimic human FDN in inducing *GLA* mutation that lead to reduced α-GalA activity [8]. However, the pathogenesis of FDN in zebra fish is different to that in humans, because Gb-3 synthase is absent in zebrafish, so deposits of Gb-3 are not involved in FDN development.

Meanwhile, during the past decade, advances in reprogramming technology for human induced pluripotent stem cells (hiPSCs) have enabled the creation of specific organ tissues called organoids. [9–11] The increasingly refined technology to differentiate various types of organoids using patient derived or genetically engineered hiPSCs has far-reaching implications for understanding disease pathophysiology, identifying disease-causing genes, and developing more precise therapeutics [12, 13]. Various protocols to develop organoids from hiPSCs have also been introduced for kidney diseases. Generated kidney organoids contain segmented structures with podocytes, proximal tubules, and distal tubules in nephron like arrangements. They can be utilized to model genetic disease mechanisms in the native genomic context and cell-type heterogeneity within the kidney [14–17]. Kidney organoids and CRISPR-Cas9 mediated knock out of *GLA* gene have also been attempted for modeling of FDN. They show Gb-3 deposition and activation of inflammatory pathway as in FDN [18]. However, hiPSC and organoid model with complete silencing of *GLA* gene cannot represent diverse manifestations of FD patients according to various mutation types. To establish a patient-tailored platform, it is necessary to determine whether hiPSCs and organoids derived from patients could represent FD phenotypes.

Based on the above background, the aim of this study was to generate hiPSCs from two male patients diagnosed as FD who showed different types of *GLA* mutation (classic vs. non-classic) in an attempt to model FDN by differentiating these cells into kidney organoids using a well-established differentiation protocol. Therefore, we intended to determine the possibility of using kidney organoids system as a patient-tailored platform for FD.

## Materials and methods

### Reprogramming of peripheral blood mononuclear cells

Peripheral blood mononuclear cells (PBMCs) were obtained from a patient with FDN. We generated hiPSCs (CMC-Fb-001 and CMC-Fb-003) as described in previous studies [19–22]. Briefly, PBMCs were isolated by centrifugation using Ficoll-Paque PLUS (GE Healthcare, Chicago, IL, USA) and cultured in StemSpa ACF (Stem Cell Technologies, Vancouver, Canada) supplemented with StemSpan™ CC110 for 4 days. Mononuclear cells were then transferred to a 24-well plate manually coated with recombinant human vitronectin (BD BioCoat). Sendai virus (SeV) (CytoTune-hiPSC 2.0 Reprogramming Kit; Life Technologies, Invitrogen, Carlsbad, CA, USA) was then added at a multiplicity of infection of three. The medium was changed daily until hiPSC colonies formed. After manual picking, hiPSC lines were maintained on vitronectin (Life Technologies, Invitrogen)-coated plates in TeSR-E8 medium (Stem Cell Technologies). On day 12 after transduction, emerging hiPSC colonies were picked individually and expanded for characterization. From day 3 to day 21 after transduction, cells were cultured in a 37 °C incubator with 5% CO_2_. We regularly checked the existence of mycoplasma in cells, which showed no positive results. (Additional file 1: Fig. S1).

### Sequencing of *GLA* gene

A high-throughput NGS assay for *GLA* gene was performed by SLC Co., Ltd. The protocol was described in our previous report [23]. Briefly, *GLA* gene was amplified using PCR. Library preparation and sequencing were performed using a Big Dye terminator v1.1 cycle sequencing kit (Applied Biosystems, Foster City, CA, USA). After sequencing runs were completed, data were aligned with those of the human reference genome sequence (NM_000169.2). Variants detected in the *GLA* gene by NGS were resequenced using the Sanger method.

### Karyotype analysis

Karyotypes of hiPSCs and peripheral blood from patient were analyzed using GTG-banding at 550 band resolution (GenDix, Seoul, Korea).

### Short Tandem Repeat analysis

STR analysis for PBMCs and hiPSCs from CMC-Fb-003 (Additional file 1: Fig. S2) was carried out using AmpFlSTR Identifiler PCR Amplification Kit 16 markers (D8S1179, D8S1179, D7S820, CSF1PO, D3S1358, TH01, D13S317, D16S539, D2S1338, Amelogenin, D5S818, FGA, D19S433, vWA, TPOX, D18S51) on a 3730/3730xl DNA Analyzer with GeneMapper (Thermo Fisher Scientific) by Cosmo Genetech, Seoul, Korea.

### Tri-lineage differentiation

For trilineage differentiation, a StemMACS™ Trilineage Differentiation Kit, human (Miltenyi Biotec) was used. Putative hiPSCs were cultivated for seven days in three different chemically defined media, driving differentiation into three germ layers. The three germ layers differentiated were fixed with 4% paraformaldehyde and stained with the following antibodies: PAX6 (1:20, Santa Cruz Biotechnology), SM22A (1:40, Santa Cruz Biotechnology), and FOXA2 (1:50, Santa Cruz Biotechnology).

### Flow cytometry

Reprogrammed hiPSCs (CMC-Fb-003) colonies were dissociated using Tryple Express (Life Technologies) and washed with PBS containing 10% FBS. Cell suspension was then stained with stage-specific embryonic antigens SSEA-4 (813-70, 1:100; Santa Cruz Biotechnology) or TRA-1-81 (TRA-1-81, 1:100; Santa Cruz Biotechnology) surface antibodies for 30 min. Intracellular staining for NANOG (1E6C4, 1:100; Santa Cruz Biotechnology) was performed by sequential incubations with fixation and permeabilization solutions (A and B Fix & Perm Solutions, Invitrogen, BD BioScience). These cells were incubated with FITC-conjugated secondary antibody (BD BioScience). Appropriate isotype controls were used for gating purposes (IgG3 isotype control to FITC (SSEA-4), IgM isotype control to FITC (TRA-1-81), IgG1 isotype control to FITC (NANOG)). Cells were analyzed using a FACS Canto II flow cytometer (BD Biosciences). Data were analyzed using a FlowJo software (TreeStar Inc., OR, USA).

### Kidney organoid differentiation from hiPSCs

**Fig. 1:**
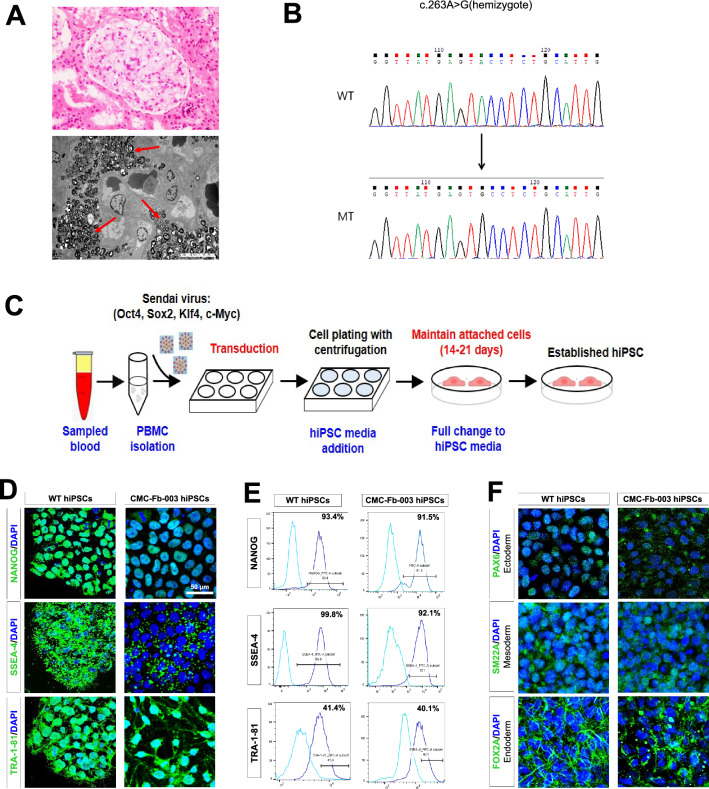
Generation of hiPSCs using peripheral blood mononuclear cells derived from patients with Fabry disease nephropathy. **A** Light and electron microscopic findings of renal tissue biopsies from FD patients. On microscopic examination, there are some vague vacuolizations (black arrow) of podocytes (upper figure, H&E stain, ×400). Multi-lamellated myelin figures (red arrow), so-called zebra bodies as typical findings of FDN, are found in electron microscopy (lower figure). **B** Sequencing results of mutation in *GLA* gene from FD patients. **C** Schematic of iPSC reprogramming from patient by Sendai virus. **D** Immunofluorescence staining of three stem cell proteins (NANOG, SSEA-4, TRA-1-81). Scale bar = 50 μm, **E** Flow cytometry analysis of pluripotency markers (NANOG, SSEA-4 TRA-1-81) in cells. **F** Immunofluorescence staining of three germ layer markers: PAX4 for detecting ectoderm differentiation, SM22A for detecting mesoderm differentiation, and FOX2A for detecting endoderm differentiation. Scale bar = 50 μm. hiPSC, human pluripotent stem cell; FDN, Fabry disease nephropathy

All wild-type (WT) hiPSC (WTC-11) and CMC-Fb-001 and CMC-Fb-003 hiPSCs were differentiated into kidney organoids following the previously published protocol [10, 16, 17] (Fig. 1B). Briefly, hiPSCs were plated in mTeSR1 medium (STEMCELL Technologies) supplemented with 10 μM Y-27632 (Biogems,1293823) onto 24-well plates pre-coated with 1.25% Corning Matrigel^®^ hESC Qualified Matrix. After 24 h, the medium was exchanged to 2.5% of Matrigel^®^ in mTeSR1. On the fourth day, the medium was replaced with Advanced RPMI (Gibco) supplemented with 12 µM CHIR-99021 (STEMCELL Technologies). Approximately 36 h later, the medium was changed to Advanced RPMI with B27 supplement (Gibco). Organoids were cultured in this medium until collection on day 21.

### Confocal microscopic analysis

hiPSCs or kidney organoids were fixed with 4% paraformaldehyde. The following antibodies were used: NANOG (1E6C4, 1:100; Santa Cruz Biotechnology), SSEA-4 (813-70, 1:100; Santa Cruz Biotechnology), TRA-1-81 (TRA-1-80, 1:100; Santa Cruz Biotechnology), biotinylated Lotus Lectin (LTL, B-1323,1:100; Vector Laboratories), E-Cadherin (ab11512, 1:50; Abcam), CD77 (551352, 1:100; BD Biosciences), and Podocalyxin (BAF1658, 1:100; R&D Systems). Antibody staining was visualized using the following secondary antibodies: Alexa Fluor 488-donkey anti-rat IgG (1:250; Invitrogen), Alexa Fluor 488-donkey anti-mouse IgG (1:250; Invitrogen), Alexa Fluor 647-donkey anti-goat IgG (1:250; Invitrogen), or Cy3-streptavidin (1:1000; Jackson ImmunoResearch). Cell nuclei were then counterstained with 4,6-diamidino-2-phenylindole (DAPI; Roche, Mannheim, Germany). Stained sections were observed using a confocal microscope (LSM700; Carl Zeiss Co. Ltd., Oberkochen, Germany). Images were converted to TIFF format and contrast levels were adjusted using Adobe Photoshop v. 13 (Adobe System, San Jose, CA, USA).

### Immunofluorescence analysis

Kidney organoids were washed in PBS, fixed with 4% paraformaldehyde for 15 min at room temperature, and washed three times with PBS. Fixed organoid cultures were blocked in 5% donkey serum (Abcam, Cambridge, UK) + 0.3% Triton-X100 (Sigma, St Louis, United States) in PBS for 60 min at room temperature and then incubated with primary antibodies in 3% BSA/PBS overnight at 4 °C, washed, incubated with secondary antibodies and DAPI overnight at 4 °C in the dark. Images were acquired using a confocal microscope (Zeiss LSM 700, Germany).

The following primary antibodies were used: anti-Gb3 (BD Biosciences, United States, AB_2738333, 1:100 dilution), anti-LTL (Vector Laboratories, United States, B-1325, 1:1000 dilution), anti-Podocalyxin (R&D systems, AF1658, 1:100 dilution), and anti-E-cadherin (BD Biosciences, United States, AB_397581, 1:100 dilution). The percentage of Gb-3-infested area in each region was calculated using the polygon program. A whole picture was assessed and graded using an open-source biological-image analysis system (ImageJ-64 bit for Windows, NIH, USA).

### Measurement of globotriaosylceramide

The concentration of Gb-3 in each hiPSCs and kidney organoid was measured by liquid chromatography-tandem mass spectrometry (LC–MS/MS). Samples were analyzed using Agilent 1200 series (Agilent, palo Alto, CA, USA) and API 4000 (SCIEX, Foster City, CA, USA). Each sample diluted in water (1:4 ratio) was added to the IS solution (N-Heptadecanoyl ceramide trioxoside, Matreya. Inc.) with 80% of dioxane, followed by mixing and centrifugation. After centrifugation, 5 μL of each sample was analyzed with the LC–ESI–MS/MS–MRM mode: m/z 1046.6 → 884.6 for C16:0 of GB-3, m/z 1174.6 → 912.7 for C18:0 of GB-3, m/z 1102.7 → 940.7 for C20:0 of GB-3, m/z 1128.7 → 966.7 for C22:1 of GB-3, m/z 1130.7 → 968.7 for C22:0 of GB-3, m/z 1156.7 → 994.7 for C24:1 of GB-3, m/z 1158.7 → 996.7 for C24:0 of GB-3, m/z 1174.9 → 1012.9 for C24:0-H of GB-3 and m/z 1060.6 → 898.6 for GB-3-IS.

### Measurement of α-galactosidase A enzyme activity

Alpha-galactosidase activity was determined using a kit (Abcam, Cambridge, UK) according to the manufacturer’s instructions. In brief, cell lysates were incubated with 5 mM 4-methylumbeliferyll-alpha-d-galactophyranoid (4mu-α-Gal) and terminated with 0.5 M glycine buffer (pH 10.3). The release of 4-methylumbelliferone (4 MU) was determined by measuring fluorescence intensity (Ex365/Em450) using a SpectraMax Gemini XS Microplate Spectrofluorometer (Molecular Device, Sunnyvale, CA, USA). Enzyme activity was shown as pmol 4MU (pmol/hr/mg) released per hour of test incubation time per mg total protein.

### Western blot analysis

hiPSCs were homogenized in boiling lysis buffer (1% sodium dodecyl sulfate [SDS], 1 mM sodium orthovanadate, and 10 mM Tris, pH 7.4). Protein concentration was determined with a BCA Protein Assay Kit (Pierce Biotechnology Inc., Waltham, MA, USA). Equal amounts of proteins were separated on an SDS–polyacrylamide gel and transferred onto a nitrocellulose membrane. For immunodetection, blots were incubated overnight in PBS containing 0.1% Tween-20 and 5% skim milk with galactosidase alpha antibody (GTX101178, 1:500; GeneTex Inc, Irvine, CA, USA). After washing with PBS containing 0.1% Tween-20, blots were then incubated with a secondary antibody conjugated to horseradish peroxidase (Jackson ImmunoResearch Laboratories, West Grove, PA, USA). After washing with PBS containing 0.1% Tween-20, blots were visualized using a western blotting luminol reagent kit (Santa Cruz Biotechnology, Santa Cruz, CA, USA).

### Electron microscopy analysis

Kidney organoid samples were fixed with 4% paraformaldehyde and 2.5% glutaraldehyde in 0.1 M phosphate buffer overnight at 4 °C. Next, samples were post-fixed with 1% osmium tetroxide in the same buffer for 1 h at 4 °C. Next, samples were dehydrated in a series of graded ethyl alcohol solutions, exchanged through acetone, and embedded in Epon 812. Ultrathin Sects. (70 to 80 nm) were cut using an ultramicrotome (Leica Ultracut UCT, Wetzlar, Germany). Ultrathin sections were double-stained with uranyl acetate and lead citrate and examined under a transmission electron microscope (JEM 1010, JEOL, Tokyo, Japan) at 60 kV. For quantitative analysis, 20 low-magnification (×6000) fields were randomly selected from each section of the cortex and the number of autophagosomes per 100 μm^2^ was determined.

### Mycoplasma monitoring

Mycoplasma analysis was performed using an e-Myco VALiD Mycoplasma PCR Kit (iNtRON Biotechnology) according to the manufacturer’s protocol. hiPSCs were cultured in a 6-well plate and cell lysate was used as the test sample to screen for mycoplasma contamination.

### Statistical analysis

All data are presented as mean ± standard error. Unpaired *t* tests or one-way analysis of variance (ANOVA) followed by the Bonferroni post hoc test was used for comparing different groups. Differences with P-values less than 0.05 were considered significant. All statistical analyses were conducted using GraphPad Prism version 5 (GraphPad Software, Inc., San Diego, CA, USA).

## Results

### Genetic mutations and clinical records for Fabry disease patient

Table 1 summarizes the clinical records of patients (CMC-Fb-001 and CMC-Fb-003) who donated PBMCs for this study. Clinical and genetic data of CMC-Fb-001 and CMC-Fb-003 have been reported previously [24, 25]. Briefly, CMC-Fb-001 was a male who showed clinical manifestations of FD such as proteinuria (473 mg/24 h), left ventricular hypertrophy (LVH), cornea verticillata, angiokeratoma, and chronic neurotic pain. Leukocyte α-GalA was reduced and *GLA* gene sequencing showed a frameshift deletion mutation [c.969delC (p.Leu324Trpfs∗24)] in exon 6 known to be a classic mutation type [24]. CMC-Fb-003 was also a male FD patient who took percutaneous kidney biopsy because of proteinuria. Biopsy finding showed swollen glomerular epithelial cells with somewhat bubbly appearance and vague vacuoles. Electron microscopy revealed typical electron-dense multi-lamellar inclusions, the so called “zebra bodies” in the cytoplasm of epithelial cells (Fig. 1A). He did not show other clinical manifestations of FD. He showed decreased leukocyte α-GalA activity. *GLA* gene sequencing showed a frameshift mutation [c.263A > G (p.Tyr88Cys)] in exon 2 (Fig. 1B) known to be a late onset renal variant type [25].

**Table 1:** Genetic mutations and clinical records of 2 fabry disease patients

	CMC-Fb-001	CMC-Fb-003
Gender	Male	Male
Age at diagnosis	32	24
GLA Mutation c	c.969delC	c.263A > G
GLA mutation p	p.Leu324Trpfs∗24	p.Tyr88Cys
Leukocyte α-galactosidase (nmol/h/mg protein)(reference range 35–100)	2.2	2.2
Gb-3 (μg/mL, normal range 3.9–9.9 μg/mL)	14.1	12.3
LysoGB-3 (ng/mL, normal range ≤ 1.74 ng/mL)	Not checked	3.52
Proteinuria (mg/24 h)	473	871
LVH on ECG	( +)	(−)
TTE	LVH	N/A
Angiokeratoma	( +)	(−)
Cornea verticillata	( +)	(−)
Anhidrosis	( +)	(−)
Chronic neurotic pain	( +)	(−)
Brain involvement	( +)	(−)

*Gb-3* globotriaocylceramide; *ECG* electrocardiography; *TTE* transthoracic echocardiography; *LVH* left ventricular hypertrophy; *N/A* Not available

### Generation of Fabry disease patients-specific human induced pluripotent stem cells

We have already generated CMC-Fb-001 and reported it previously [20]. Additionally, we generated hiPSCs (CMC-Fb-003) using PBMCs isolated from the patient (Fig. 1B). The mutation (c.263A > G) detected in the patient’s blood was also detected in the *GLA* gene of CMC-Fb-003 hiPSCs (Fig. 1C). In addition, CMC-Fb-003 showed typical hiPSC colony formation and normal karyotyping (Additional file 1: Fig. S3A, S3B). Pluripotency-associated markers NANOG, SSEA4, and TRA-1–81 on hiPSCs were successfully detected by confocal microscopy (Fig. 1D) and flow cytometry (Fig. 1E) in WT and CMC-Fb-003. Tri-lineage differentiation assays demonstrated that WT and CMC-Fb-003 hiPSCs were successfully differentiated into ectoderm, mesoderm, and endoderm (Fig. 1F).

### Comparison of expression levels of α-GalA protein and accumulation of Gb-3 in hiPSCs

**Fig. 2:**
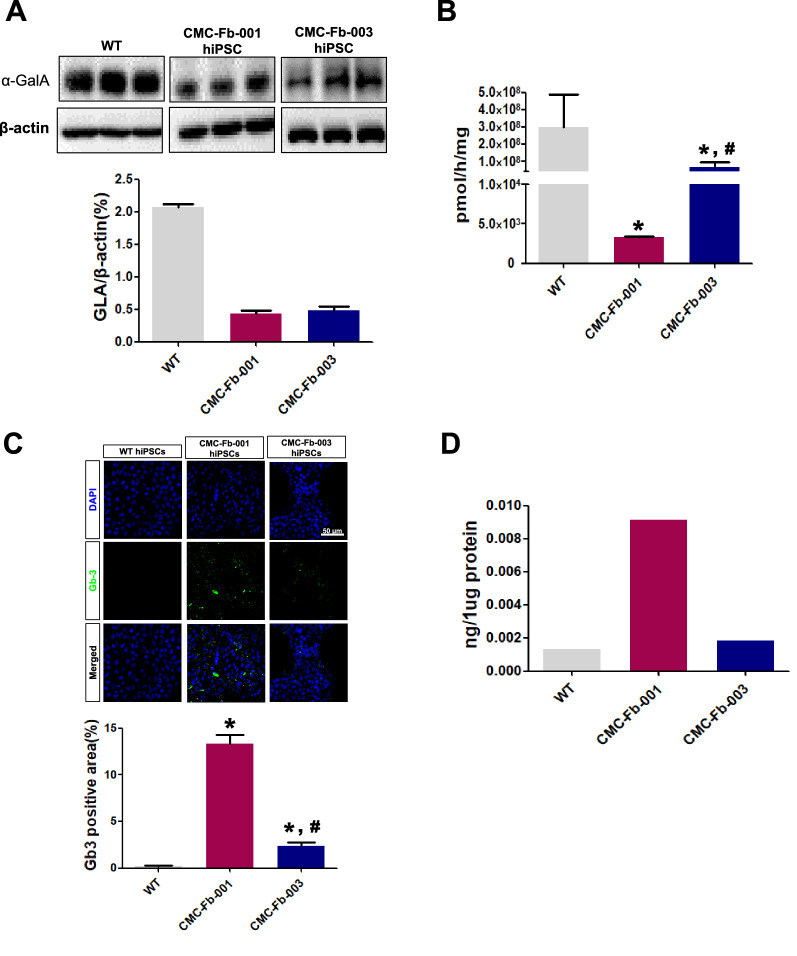
Comparison of α-GalA expression, α-GalA activity, and amount of Gb-3 deposition in hiPSC cells by immunofluorescent study and LC–MS/MS. **A** Representative Western blot and quantification graphs of the expression of α-GalA in hiPSC. **B** Quantification of α-GalA activity analysis. **C** Immunofluorescence staining with an antibody against Gb-3 in WT, CMC-Fb-001, and CMC-Fb-003. **D** Quantification of Gb-3 level in hiPSCs. Mean values from three independent experiments are shown with standard deviation error bars. α-GalA, α-galactosidase A; Gb-3; globotriaocylceramide, WT, wild-type; hiPSC, human induced pluripotent stem cells

When the amount of α-GalA protein and the α-GalA enzyme activity were compared, α-GalA expression in western blot (WT 206.6% ± 5.5% vs. CMC-Fb-001 43.4% ± 4.3% vs. CMC-Fb-003 35.6% ± 1.2%, respectively; *p* < 0.05 vs. WT) (Fig. 2A) and activity measured by enzyme assay (WT 3 × 10^8^ ± 1.9 × 10^8^ pmol/h/mg, CMC-Fb-001 3.3 × 10^3^ ± 1.2 × 10^2^ pmol/h/mg, CMC-Fb-003 6.5 × 10^7^ ± 3.3 × 10^7^ pmol/h/mg, respectively; *p* < 0.05 vs. WT) (Fig. 2B) were significantly decreased in CMC-Fb-001 and CMC-Fb-003 in comparison with those of WT. In IF staining, Gb-3 was detected in CMC-Fb-001 and CMC-Fb-003 but not in WT hiPSCs (WT 0.2% ± 0.08% vs. CMC-Fb-001 13.32% ± 0.95% vs. CMC-Fb-003 2.34% ± 0.44%, respectively; *p* < 0.05 vs. WT) (Fig. 2C). Amount of Gb-3 deposition within hiPSCs measured by LC–MS/MS was significantly increased in CMC-Fb-001 (9.1 × 10^–3^ ng/μg of protein) compared to that in CMC-Fb-003 (1.8 × 10^–3^ ng/μg of protein) or WT hiPSC (2.3 × 10^–3^ ng/1 μg of protein) (Fig. 2D).

### Differentiation of WT and FD-patients specific hiPSCs into kidney organoids

**Fig. 3:**
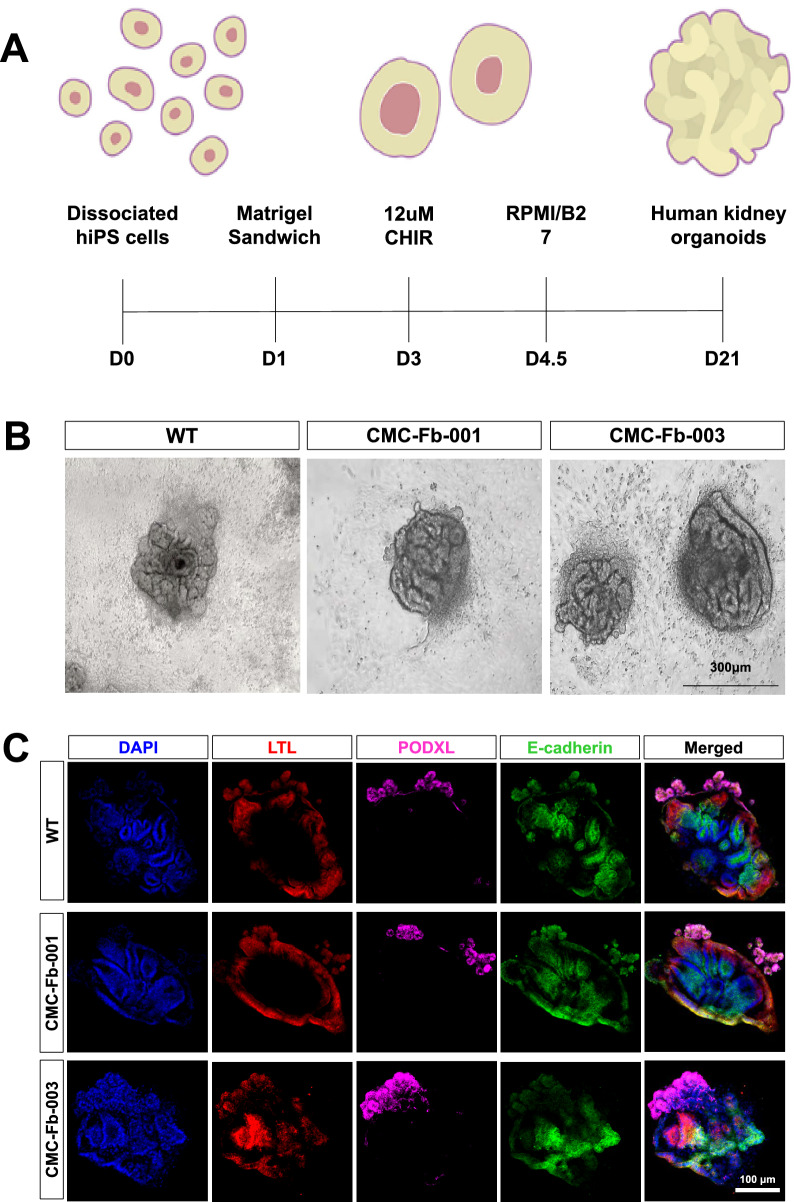
Immunohistochemistry of nephron marker in kidney organoids. **A** Schematic timeline of kidney organoid differentiation protocol. Media and supplements used throughout the protocol are indicated. **B** Representative brightfield image of kidney organoids. Scale bar = 200 μm. **C** Representative immunofluorescence staining of LTL, PODXL, E-cadherin in WT, CMC-Fb-001, and CMC-Fb-003 kidney organoids. Scale bar = 100 μm

For modeling of FD within a kidney context, we differentiated all WT and CMC-Fb-001 and CMC-Fb-003 into kidney organoids using an adherent culture protocol previously reported (Fig. 3A) [14, 16, 17]. At 21 days after plating, typical segmented tubular structures were detected (Fig. 3B). In IF staining and examination under confocal microscopy, all WT, CMC-Fb-001, and CMC-Fb-003 were successfully differentiated into kidney organoids without major structural differences in expression levels of markers of nephron structure (including PODXL in glomerular epithelial cells, LTL in the proximal tubule, and E-cadherin in the distal tubule) in appropriately patterned and contiguous segments (Fig. 3C).

### Comparison of α-GalA activity and deposition of Gb-3 in kidney organoids

**Fig. 4:**
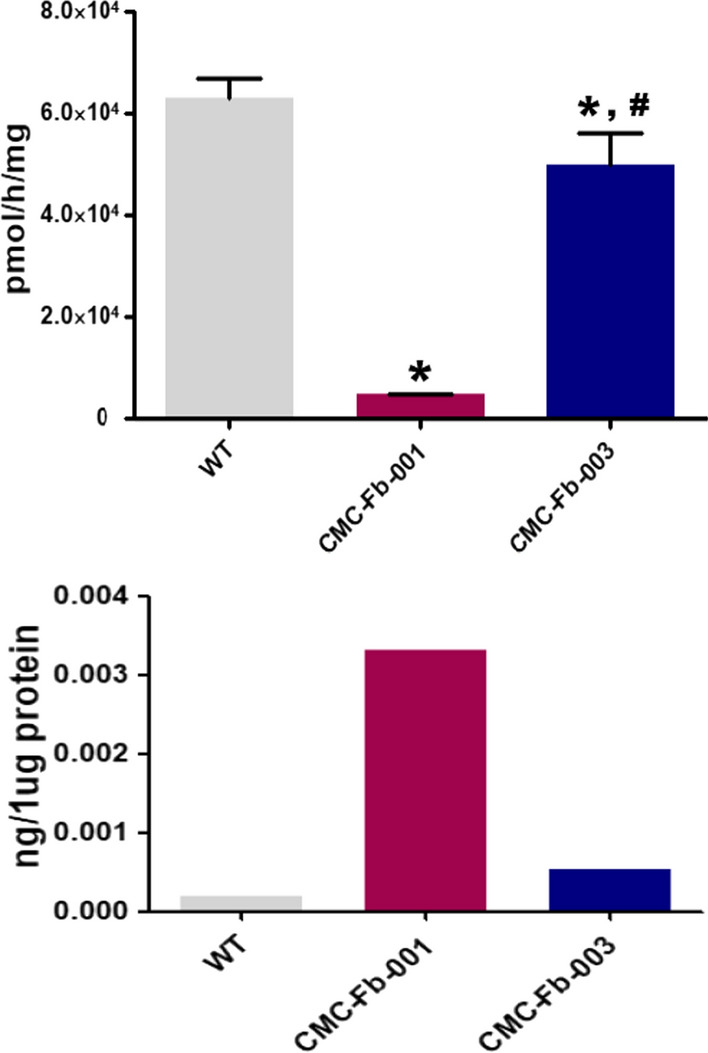
Comparison of α-GalA activity and amount of Gb-3 deposition in kidney organoids by LC–MS/MS. **A** Quantification of α-GalA activity analysis. **B** Quantification of Gb-3 level in kidney organoids by LC-MC/MC. Mean values from three independent experiments are shown with standard deviation error bars. Gb-3; globotriaocylceramide; α-GalA, α-galactosidase A enzyme

**Fig. 5:**
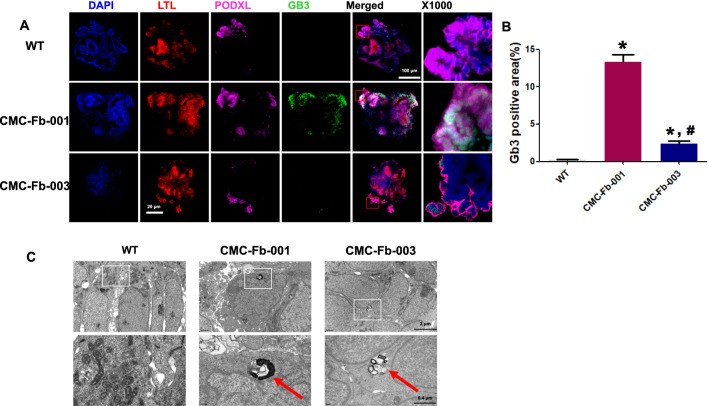
Comparison of Gb-3 deposition and zebra body in kidney organoids. **A** Representative immunofluorescence staining of Gb3, LTL, and PODXL in WT, CMC-Fb-001, and CMC-Fb-003 kidney organoids. Scale bar = 100 μm. Enlarged image at the podocyte is shown. Scale bar = 20 μm. **B** Quantification of the percentage of Gb-3 positive area. **C** Raw and enlarged TEM images of WT, CMC-Fb-001, and CMC-Fb-003 kidney organoids. Red arrows indicate lysosomal Gb-3 depositions. Scale bar = 2 μm for upper figures in **C**. Scale bar = 0.4 μm for lower figures in **B**. Gb-3; globotriaocylceramide

At the same time as differentiated kidney organoids were expressing markers of nephron structures, we also analyzed enzymatic activity of α-GalA and compared the amount of Gb-3 deposition. Activity of α-GalA in CMC-Fb-001 kidney organoids was the lowest. It was lower in CMC-Fb-003 than in WT but higher than in WT kidney organoids (CMC-Fb-001 4.6 × 10^3^ ± 1.4 × 10 vs. WT 6.3 × 10^4^ ± 3.9 × 10^3^ vs. CMC-Fb-003 4.9*10^4^ ± 6.4 × 10^3^, p < 0.05 vs. WT) (Fig. 4A). In IF staining to observe deposition of Gb-3 in kidney organoids, we could detect the staining for Gb-3 within kidney organoid. It especially overlapped by PODXL in both FD-patients derived kidney organoids. It was more significant in CMC-Fb-001 than in CMC-Fb-003. Gb-3 deposit was not detected in WT kidney organoids (WT 0.2% ± 0.1% vs. CMC-Fb-001 13.3% ± 1% vs. CMC-Fb-003 2.3% ± 0.4%, respectively; *p* < 0.05 vs. WT) (Figs. 5A, B). Gb-3 level measured by LC–MS/MS in kidney organoids was the highest in CMC-Fb-001 (3.3 × 10^–3^ ng/μg), followed by that in CMC-Fb-003 (5.3 × 10^–4^ ng/μg of protein). It was the lowest in WT organoids (1.8 × 10^–4^ ng/μg) (Fig. 5B). Multi-lamellar intracellular structures were observed by EM in both FD-patients kidney organoids, but not in WT kidney organoids (Fig. 5C).

## Discussion

In this study, we generated FD-patient derived hiPSCs and differentiated them into kidney organoids. In comparison with WT kidney organoids, FD-patient derived hiPSCs and kidney organoids showed decreased α-GalA activity and increased deposition of Gb-3. Such FD-phenotype was more severe in CMC-Fb-001 than in CMC-Fb-003. Our study suggests that kidney organoids generated using FD-patient hiPSCs can recapitulate the phenotype of FDN. It might also represent the severity of FD according to the mutation type.

Our first aim was to investigate whether patient derived hiPSCs or kidney organoids could show the phenotype of FD. Kidney organoids from genetically modified or patient derived hiPSCs, successfully recapitulated the phenotype of various genetic kidney disease [14–17, 26]. CMC-Fb-001 and CMC-Fb-003 patients were diagnosed as FD by *GLA* gene sequencing. They showed manifestations of FD [24, 25]. To investigate FD phenotype in hiPSCs or kidney organoids, we can explore the activity of α-GalA and the deposition of Gb-03. According to the characteristics of FD, the simultaneous comparison of the activity of α-GalA and the accumulation of Gb-03 is more helpful to determine whether the model in this experiment mimics the process of FD in humans [27, 28]. In this study, the activity of α-GalA and α-GalA protein amount were significantly decreased and the deposition of Gb-3 was significantly increased in CMC-Fb-001 and CMC-Fb-003 in comparison with those in WT in both hiPSCs and kidney organoids. In kidney organoids, zebra body formation was detected in both FD kidney organoids, but not in WT organoids. Interestingly, in the IF study, detection of Gb-3 deposition was mostly overlapped with PODXL, a podocyte marker. This finding is consistent with a previous study showing that Gb-3 deposition is dominant especially in podocytes in kidney tissues of FDN patients [29]. Taken together, results of our study showed that FD-patients derived hiPSCs or kidney organoids could represent FDN.

Of note, the severity of FD phenotype in terms of Α-GalA enzyme activity and Gb-3 deposition was more severe in CMC-Fb-001 than in CMC-Fb-003 hiPSC and kidney organoids. Different mutations in *GLA* can produce different effects on α-GalA. Therefore, the spectrum of clinical presentations in FD male patients can vary widely, ranging from a severe classic phenotype to a relatively mild non-classic phenotype [30, 31]. In classic male FD patients, intracellular deposition of Gb-3 initiates at childhood and accumulation of Gb-3 in blood vessels and capillaries with increasing age can lead to heart failure, progressive kidney disease, and stroke [32, 33]. Non-classic phenotype comprise almost 70% of FD patients [34]. Relatively higher activity of α-GalA allows Gb-3 to accumulate in tissues at a slow rate with organ damage developing at a relatively late age in comparison with classic FD [31, 32, 34]. CMC-Fb-001 was from a patient with a classic type of FD, while CMC-Fb-003 was from a patient with a non-classic type of FD [24, 25]. Such mutational difference might have led to different manifestations of the disease phenotype at the cellular level.

CMC-Fb-001 hiPSC showed lower expression of α-GalA protein and lower activity of α-GalA than WT hiPSCs. In contrast, CMC-Fb-003 showed lower expression of Α-GalA protein but relatively intact activity of α-GalA. Usually, the amount of α-GalA protein and enzymatic activity of α-GalA show a significant correlation. However, previous reports have shown decreased expression of α-GalA protein or RNA even though the activity of α-GalA is within normal range. This can have occurred because RNA is inherently unstable [35, 36]. Therefore, in this study, even though the activity of α-GalA did not change much compared with the WT group, the lower amount of α-GalA protein in CMC-Fb-003 can have led to an accumulation of Gb-3. In clinical data, CMC-Fb-003 patient showed decreased activity of α-GalA in contrast with results of this study. However, this patient was already an adult when he was diagnosed as FD. hiPSC and kidney organoid represent a very young age state. Therefore, it was possible that the activity of α-GalA was normal in childhood of CMC-Fb-003 patient as in CMC-Fb-003 hiPSCs and kidney organoids. Another possible reason for this discrepancy might be that the genotype–phenotype correlation is not perfect in FD because environmental factors can affect the disease severity and phenotypic variability of FD [33]. However, hiPSC and kidney organoids system cannot represent environmental factors. They can just represent the effect of germline mutation. Further investigation may be required to clarify this issue.

Results of this study can be used for various purposes. More than 965 mutations in the *GLA* gene could cause FD [37]. There are still many mutations, of which pathologic significance has not been clarified [38]. Currently used experimental models to functionally validate genetic variants associated with FD are far from perfect [39]. It has been already reported that the severity of disease recapitulated in kidney organoids can be different according to the pathogenicity of mutation type in genetic kidney disease [40]. Patient derived hiPSCs appear to provide a unique platform to study genetic mechanisms and also functional or clinical significance of FD as they retain all genetic information of the original affected individuals. Moreover, patient derived hiPSCs can contain the epigenetic changes which can affect the accessibility of the *GLA* gene and alter the level of α-GalA activity [41–43].

Indeed, for disease modeling, although gene editing in WT hiPSCs has the advantage in complete gene silencing, patient-derived hiPSCs are more relevant to patient and mutation type-specific modeling. Thus, using our platform, first we can generate patient derived hiPSCs using somatic cells isolated from patients. Second, we can differentiate them into kidney organoids. Third, we can confirm whether the mutation is associated with the disease phenotype. Therefore, we can confirm the significance of mutation.

This study has some limitations. Because of the rarity of FD and the complex task to make patient derived hiPSCs and kidney organoids, we only included two patients in this study. If more convenient method to develop hiPSCs and kidney organoids becomes available, we can include more patients in this study and investigate the relationship between mutation type and disease severity in more detail. Second, kidney organoids represent immature form of kidney. Considering that overt FDN usually develops in adulthood, there can be an argument that kidney organoids may not be appropriate for modeling of FDN [44]. Lastly, unfortunately, lyso-GB-3 did not show increase in patients derived kidney organoids (Additional file 1: Fig. S4) even though serum level lyso-Gb-3 is known to be an ideal biomarker for the diagnosis of FD in clinical practice. The origin of lyso-Gb-3 is still unclear. It can be synthesized by sequential glycosylation of accumulating sphingolipid precursors or originate from deacylation of stored Gb3 [45]. The potential role of this metabolite in the manifestation of the disease deserves further investigation.

In summary, we found that FD phenotype was successfully recapitulated in FD patient-specific hiPSCs and kidney organoids. Enzymatic activity of α-Gal A was significantly decreased, whereas GB-3 level measured by LC–MS.MS and GB-3 deposits analyzed in IF staining images were significantly increased in FD-patient hiPSCs and kidney organoids in comparison with those in WT. Typical zebra bodies were detected in both FD-patient derived kidney organoids. Moreover, disease severity was significantly different according to the mutation type. It was more significant in hiPSCs and kidney organoids derived from a classic FD male patient than in those from a non-classic type. In conclusion, FD-patients derived kidney organoids may recapitulate the phenotype of FDN as a mutation specific pattern. It can be used as a platform for in vitro disease modeling and tailored medicine.

## Supplementary Information

**Additional file 1: Figure S1.** PCR results of Mycoplasma detection for CMC-Fb03 hiPSC. **Figure S2.** Cell line authentication by short tandem repeat analysis. STR analysis shows 100% match between PBMC and hiPSC line in CMC-Fb-003. STR, short tandem repeat; hiPSC, human induced pluripotent stem cells. **Figure S3.** Characterization of CMC-Fb-003 cells. **A** Typical morphology of hiPSC colony of CMC-Fb-003 hiPSC. Scale bar = 40 μm. **B** Chromosome karyotyping of CMC-Fb03 hiPSC. hiPSC, human induced pluripotent stem cells. **Figure S4.** Lyso-Gb-3 level in kidney organoids. Gb-3, globotriaocylceramide.

## Data Availability

The data that support the findings of this study are available on request from the corresponding author. The data are not publicly available due to privacy or ethical restrictions.
