# Supplementary material for: Modeling of Fabry disease nephropathy using patient derived human induced pluripotent stem cells and kidney organoid system
Source: J Transl Med. 2023 Feb 22;21:138. doi: 10.1186/s12967-023-03992-0 (PMC9948377; doi:10.1186/s12967-023-03992-0)
Supplement: Supplementary file 1 — Additional file 1: Figure S1. PCR results of Mycoplasma detection for CMC-Fb03 hiPSC. Figure S2. Cell line authentication by short tandem repeat analysis. STR analysis shows 100% match between PBMC and hiPSC line in CMC-Fb-003. STR, short tandem repeat; hiPSC, human induced pluripotent stem cells. Figure S3. Characterization of CMC-Fb-003 cells. A Typical morphology of hiPSC colony of CMC-Fb-003 hiPSC. Scale bar = 40 μm. B Chromosome karyotyping of CMC-Fb03 hiPSC. hiPSC, human induced pluripotent stem cells. Figure S4. Lyso-Gb-3 level in kidney organoids. Gb-3, globotriaocylceramide. [file 12967_2023_3992_MOESM1_ESM.docx]

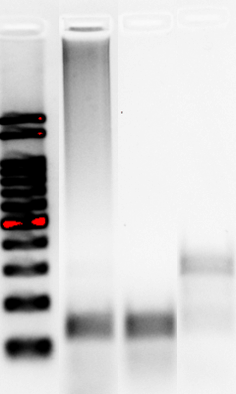


Mycoplasma

Internal control

CMC-Fb03

N

P

**Supplement figure 1.** PCR results of Mycoplasma detection for CMC-Fb-003 hiPSC.


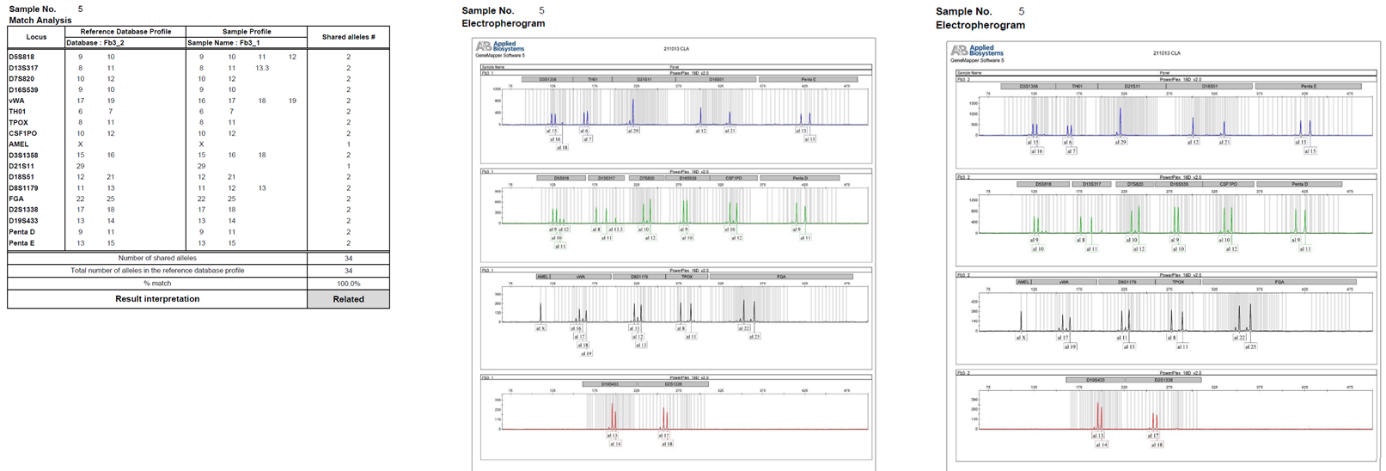


**Supplement figure 2.** Cell line authentication by short tandem repeat analysis. STR analysis shows 100% match between PBMC and hiPSC line in CMC-Fb-003

STR, short tandem repeat; hiPSC, human induced pluripotent stem cells


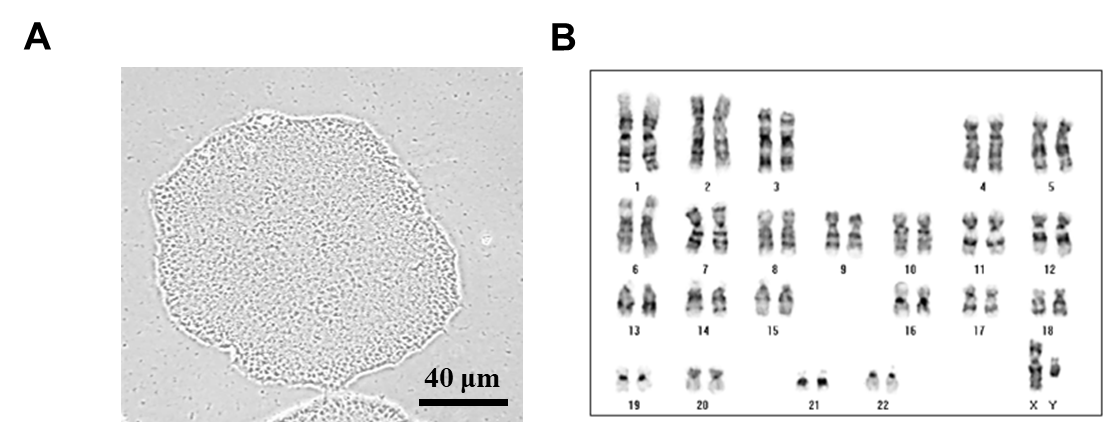


**Supplement figure 3. Characterization of CMC-Fb-003 cells. (A)** Typical morphology of hiPSC colony of CMC-Fb-003 hiPSC. Scale bar = 40μm. **(B)** Chromosome karyotyping of CMC-Fb03 hiPSC.

hiPSC, human induced pluripotent stem cells


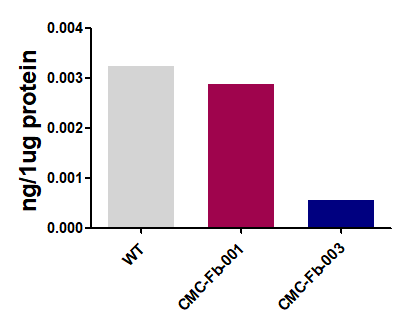


**Supplement figure 4.** Lyso-Gb-3 level in kidney organoids

Gb-3, globotriaocylceramide
